# Supplementary material for: Association of Hippocampal Subfield Volumes with Amyloid-Beta Deposition in Alzheimer’s Disease
Source: J Clin Med. 2022 Mar 10;11(6):1526. doi: 10.3390/jcm11061526 (PMC8955328; doi:10.3390/jcm11061526)
Supplement: Supplementary file 1 [file jcm-11-01526-s001.zip › jcm-1605637-supplementary.pdf]

Supplemental Table S1. Hippocampal subfield volumes in the diagnostic groups

	Hippocampal subfield volumes			*P* value - Adjusted*		
	CU	MCI	AD	CU vs MCI	CU vs AD	MCI vs AD
Total Hippo	4.66±0.55	3.64±0.91	2.91±0.80	0.065	**0.002**	1.000
CA1	1.61±0.26	1.37±0.28	1.09±0.27	0.448	**0.021**	0.802
CA2-3	0.36±0.07	0.32±0.06	0.24±0.10	1.000	0.052	0.409
CA4-DG	1.26±0.16	0.87±0.28	0.70±0.25	**0.040**	**< 0.001**	0.717
SRLM	0.91±0.13	0.58±0.27	0.42±0.18	**0.029**	**< 0.001**	0.801
Subiculum	0.53±0.09	0.50±0.12	0.48±0.16	0.380	1.000	0.092

Data are presented as mean ± SD. *Adjustment for age, sex, presence of APOE ε4, and intracranial volume (ICV). Abbreviations: CU = cognitively unimpaired, MCI = mild cognitive impairment, AD = Alzheimer's disease, Total Hippo = whole hippocampus volume, CA = cornu ammonis, SRLM = stratum radiatum/lacunosum/moleculare.

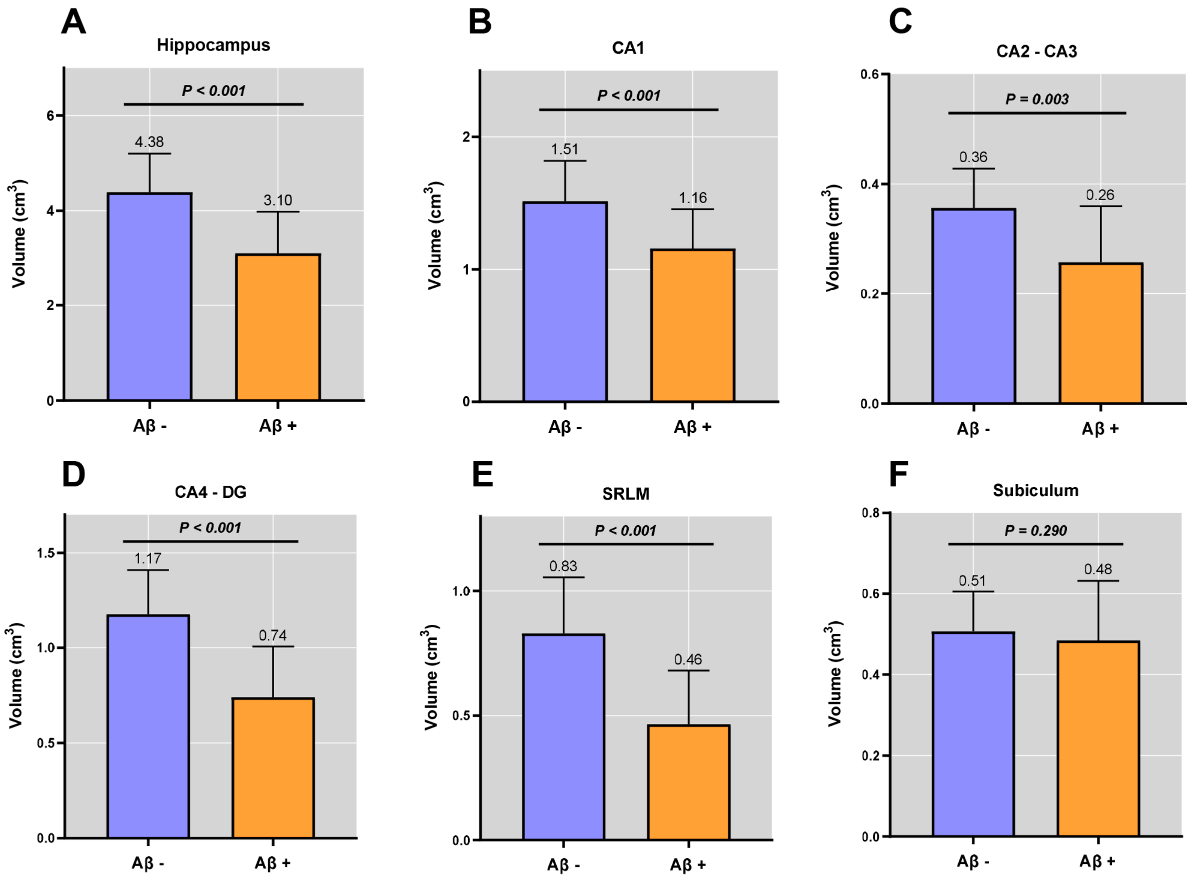

Supplement Figure S1. The hippocampal subfield volumes in Aβ+ and Aβ− participants. (A) Total hippocampal volume (B) CA1 (C) CA2–3 (D) CA4-DG (E) SRLM (F) Subiculum. Abbreviations: AD = Alzheimer's disease, CU = cognitively unimpaired, MCI = mild cognitive impairment, CA = cornu ammonis, SRLM = stratum radiatum/lacunosum/moleculare.
